# Deep Optic Nerve Head Structural Alterations in Adults with Cystic Fibrosis

**DOI:** 10.3390/jcm15114308

**Published:** 2026-06-02

**Authors:** Sławomir Liberski, Bartosz Skulimowski, Filip Waśniewski, Aleksandra Kałużna, Goran Petrovski, Szczepan Cofta, Jarosław Kocięcki

**Affiliations:** 1 Department of Ophthalmology, Poznan University of Medical Sciences, A. Szamarzewskiego 84, 60-569 Poznan, Poland; skulimowskibartosz@gmail.com (B.S.); olakaluzna2003@gmail.com (A.K.); j.kociecki@gmail.com (J.K.); 2 Doctoral School, Poznan University of Medical Sciences, Bukowska 70, 60-812 Poznan, Poland; 3 1st Department of Cardiology, University Clinical Hospital, Poznan University of Medical Sciences, 60-786 Poznan, Poland; filip.wasniewski@usk.poznan.pl; 4 Center for Eye Research and Innovative Diagnostics, Department of Ophthalmology, Institute for Clinical Medicine, University of Oslo, Kirkeveien 166, 0450 Oslo, Norway; goran.petrovski@medisin.uio.no; 5 Department of Ophthalmology, Oslo University Hospital, Kirkeveien 166, 0450 Oslo, Norway; 6 Department of Respiratory Medicine, Allergology and Pulmonary Oncology, Poznan University of Medical Sciences, A. Szamarzewskiego 84, 60-569 Poznan, Poland; s.cofta@gmail.com

**Keywords:** cystic fibrosis, optic nerve head, lamina cribrosa, optical coherence tomography, retinal nerve fiber layer, structural biomarkers

## Abstract

**Background:** Cystic fibrosis (CF) is a systemic genetic disorder characterized by chronic inflammation, hypoxia, and metabolic imbalance that may affect neural and microvascular structures. While previous studies have evaluated superficial optic nerve head (ONH) parameters in CF, deep ONH structures, particularly the lamina cribrosa (LC), remain insufficiently explored. This study aimed to assess both superficial and deep ONH morphology in adults with CF using swept-source optical coherence tomography (SS-OCT). **Methods:** This observational case–control study included 34 CF individuals and 34 age- and sex-matched healthy controls. CF patients were examined at baseline and after 12–18 months of cystic fibrosis transmembrane conductance regulator (CFTR) modulator therapy. All participants underwent comprehensive ophthalmic examination and SS-OCT imaging. Assessed parameters included peripapillary retinal nerve fiber layer (RNFL) thickness, lamina cribrosa thickness (LCT), and lamina cribrosa depth (LCD). Between-group comparisons were performed using ANCOVA adjusted for axial length (AL) and IOP. **Results:** After adjustment for AL and IOP, total RNFL thickness did not differ significantly between the CF group and controls (F(1,92) = 0.363, *p* = 0.548). However, CF patients demonstrated significantly reduced central LCT (170.2 µm [95% CI 162.2–178.2] vs. 214.6 µm [95% CI 208.6–220.7]; F(1,91) = 72.205, *p* < 0.001) without markedly altered LCD (425.5 µm [95% CI 390.4–454.2] vs. 354.7 µm [95% CI 329.6–379.8]; F(1,94) = 10.090, *p* = 0.099) compared with controls. Intraocular pressure was also higher in CF patients (17.50 mmHg [95% CI 16.60–18.41] vs. 15.87 mmHg [95% CI 15.19–16.55]; F(1,83) = 7.660, *p* = 0.007). Longitudinally, total RNFL thickness decreased from 106.5 µm (IQR 15.25) to 102.0 µm (IQR 15.5) following therapy (z = 3.488, *p* < 0.001), while LCT (*p* = 0.364) and LCD (*p* = 0.660) remained stable. **Conclusions:** CF is associated with significant alterations in deep ONH structures, characterized by thinner LC, independent of ocular biometry. In contrast, superficial RNFL differences appear to be largely influenced by AL. LC parameters, particularly LCT, may represent potential structural markers of systemic involvement in CF, pending further validation.

## 1. Introduction

Cystic fibrosis (CF) is a genetic systemic disease characterized by progressive pulmonary and gastrointestinal involvement, secondary to mutations in the CFTR gene on chromosome 7. Recent advances in medical care have significantly improved life expectancy and quality of life in CF patients [1,2]. Beyond respiratory and metabolic complications, growing evidence suggests that CF may be associated with systemic alterations related to chronic inflammation, hypoxia, and metabolic imbalance [3].

CFTR is expressed in numerous ocular tissues, including the corneal and conjunctival epithelium, corneal endothelium, non-pigmented ciliary epithelium, and retinal pigment epithelium [4]. As a chloride and bicarbonate channel, it plays a major role in ion transport and fluid secretion, maintaining epithelial hydration and ionic homeostasis within the ocular microenvironment [2,4]. CFTR dysfunction may disrupt fluid balance and tissue hydration, and may affect ocular structures by altering tissue biomechanics, vascular regulation, and metabolic homeostasis. Highly effective CFTR modulator therapy (HEMT) partially restores CFTR function at the cellular level, improving ion transport and fluid regulation [2], which may contribute to normalization of epithelial hydration and ionic balance.

The optic nerve head (ONH) is an anatomical structure localized at the interface between the eye and the central nervous system (CNS) [5]. Previous studies have shown structural alterations of the ONH in a variety of systemic diseases [6,7]. Among deep ONH structures, the lamina cribrosa (LC) plays a crucial biomechanical and vascular role, as axons pass through this structure when exiting the eye and may be affected by mechanical and vascular stress [8]. Optical coherence tomography (OCT) is a non-invasive imaging method widely used to evaluate retinal and ON structures. Recently, OCT has been shown to be useful for diagnosing and monitoring many systemic diseases, especially those involving chronic hypoxia, inflammation, and neurodegenerative processes [9]. Swept-source OCT (SS-OCT) provides improved tissue penetration and enhanced visualization of deeper ONH structures, including the LC, compared with conventional spectral-domain OCT (SD-OCT) [10].

Previous studies have detected changes in the superficial structures of the optic nerve in CF patients, particularly a sectoral decrease in peripapillary retinal nerve fiber layer (RNFL) thickness [11,12]; however, the deeper ONH structures, particularly the LC, remain largely unexplored. Given the systemic nature of CF and its known impact on neural and microvascular integrity [3,13], a detailed evaluation of ONH morphology may provide new insights into disease-related structural changes. Therefore, this study aimed to assess ONH superficial and deep morphological parameters in CF individuals using SS-OCT.

## 2. Materials and Methods

### 2.1. Study Design and Participants

This observational case–control study included adult patients with CF and age- and sex-matched healthy controls. A total of 34 CF individuals and 34 healthy individuals were enrolled. CF patients were examined at two time points: prior to the initiation of CFTR modulator therapy (elexacaftor/tezacaftor/ivacaftor; (Kaftrio®) in combination with ivacaftor (Kalydeco®), Vertex Pharmaceuticals, Boston, MA, USA) and after 12–18 months of treatment, while control subjects underwent a single ophthalmic examination. CF patients were recruited at the Department of Respiratory Medicine, Allergology and Pulmonary Oncology, and the ophthalmological examination was conducted at the Department of Ophthalmology, Poznan University of Medical Sciences, Poznan, Poland.

The study was conducted in accordance with the tenets of the Declaration of Helsinki and received approval from the Bioethics Committee of the Poznan University of Medical Sciences (approval no. 965/22). Written informed consent was obtained from all participants prior to inclusion in the study.

Inclusion criteria for the CF group were a confirmed diagnosis of CF and age ≥18 years. The exclusion criteria for all participants comprised a history of glaucoma or ocular hypertension, optic neuropathy of any etiology, retinal disease affecting the macular or peripapillary region, previous intraocular surgery, high refractive error (defined as spherical equivalent exceeding ±6.0 diopters), as well as any condition that could affect OCT image quality or ONH morphology, or the presence of uncontrolled systemic diseases known to significantly affect the optic nerve.

Demographic and clinical variables collected included age, sex, axial length (AL), intraocular pressure (IOP), and pulmonary function parameters, including forced expiratory volume in one second (FEV1).

### 2.2. Ophthalmic Examination

All participants underwent a comprehensive ophthalmic examination, including best-corrected visual acuity assessment (BCVA), slit-lamp biomicroscopy (anterior and posterior segment), intraocular pressure measurement, and ocular biometry, including AL.

### 2.3. Optical Coherence Tomography Imaging

All OCT examinations were performed using a SS-OCT device (DRI OCT Triton, Topcon Corporation, Tokyo, Japan). Image acquisition and analysis were performed using the device software (IMAGEnet 6; Topcon Corporation, Tokyo, Japan). A 3D Disc scan was used to assess of ONH morphology and peripapillary RNFL thickness, including total RNFL thickness and sectoral measurements (superior, inferior, nasal, and temporal quadrants). In addition, the inferior–superior RNFL difference was calculated as an exploratory structural parameter.

**Figure 1:**
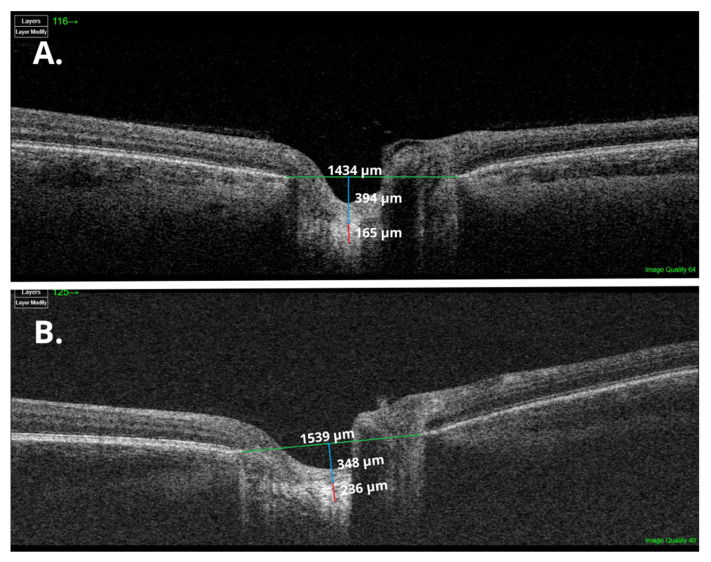
Representative SS-OCT image of the optic nerve head (ONH) demonstrating measurement methodology. The horizontal B-scan passing through the center of the ONH was used to assess lamina cribrosa thickness (LCT) (red lines) and lamina cribrosa depth (LCD) (blue lines), defined relative to the Bruch’s membrane opening (BMO) reference plane (green lines). (**A**) Eye of a patient with cystic fibrosis (CF). (**B**) Eye of an age-matched healthy control.

Deep ONH structures were assessed manually on B-scan images. Lamina cribrosa thickness (LCT) and lamina cribrosa depth (LCD) were measured using the horizontal B-scan passing through the center of the ONH. LCT was defined as the distance between the anterior and posterior borders of the LC, while LCD was measured as the perpendicular distance from the Bruch’s membrane opening (BMO) reference plane to the anterior laminar surface (Figure 1).

### 2.4. Eye Selection

Both eyes of each participant were examined. For all evaluated parameters, a preliminary analysis was performed to determine whether right (OD) and left (OS) eyes differed within each study group. No statistically significant OD-OS differences were identified in either the CF group or the control group (all *p* > 0.05) (Supplementary Table S1–4). Given the absence of inter-eye asymmetry and to avoid inflating the sample size, a single value per participant was derived by averaging OD and OS measurements.

### 2.5. Measurement Methodology and Reproducibility

Manual measurements of LC parameters were performed independently by two experienced investigators (SL and BS). Measurements were obtained using the device’s built-in caliper tool in the software (IMAGEnet 6, Topcon Corporation, Tokyo, Japan). The mean value from OD and OS was used for statistical analysis. When the inter-observer difference exceeded a prespecified threshold of 10 µm, the scan was jointly reviewed, and a consensus value was established in consultation with a senior investigator (JK).

To improve visualization of the LC borders, image display parameters, such as contrast and brightness, were adjusted as needed. In selected cases, color inversion (black-white reversal) was applied to enhance the visibility of laminar borders. All measurements were performed on grayscale B-scan images.

Only OCT scans with adequate image quality were included in the analysis. A total of 18/204 scans (8.8%) were excluded due to insufficient image quality or inadequate visualization of the LC.

### 2.6. Statistical Analysis

All statistical analyses were conducted using JASP (Version 0.95.3, JASP Team, University of Amsterdam, Amsterdam, The Netherlands). The graphs included were created using the R programming language (Version 4.5.2, University of Auckland, Auckland, New Zealand) within the Visual Studio Code environment version 1.110 (Microsoft, Washington, DC, USA).

Continuous variables were tested for normality using the Shapiro–Wilk test. Normally distributed data are presented as mean ± standard deviation (SD), whereas non-normally distributed variables are reported as median with interquartile range (IQR). Categorical variables are presented as counts and percentages.

Baseline comparisons between the CF and control groups were performed using independent-samples *t*-tests or Mann–Whitney U tests, depending on the data distribution. Categorical variables were compared using the chi-square test or Fisher’s exact test.

Between-group comparisons of OCT parameters were additionally evaluated using analysis of covariance (ANCOVA) with study group (CF vs. control) as the main factor and AL as a covariate. The assumption of homogeneity of regression slopes was verified by testing the group by AL interaction. When the interaction was not significant, it was removed, and the final ANCOVA model was calculated. Effect sizes were reported as partial eta-squared (ηp^2^), and adjusted means were expressed as estimated marginal means with 95% confidence intervals. Longitudinal changes within the CF group between baseline and follow-up examination after 12–18 months of CFTR modulator therapy were evaluated using the Wilcoxon signed-rank test. Correlation analyses were performed to assess relationships between OCT-derived parameters and selected clinical variables, including AL and pulmonary function (FEV_1_), using Pearson or Spearman correlation coefficients as appropriate. A two-sided *p*-value < 0.05 was considered statistically significant.

## 3. Results

### 3.1. Characteristics of the Study Participants

Baseline demographic, clinical, and ocular characteristics are summarized in Table 1. Compared with controls, patients with CF differed in AL and IOP, while age and sex distribution were comparable between groups.

As the AL and IOP significantly differed between the CF and control group, we performed ANCOVA analysis to further examine the significance of each comparison. First, we tested the group × AL and group × IOP interactions for each outcome. These terms were not significant in all models (all *p* > 0.05), therefore, they were removed and the final analyses were performed using ANCOVA models including group (CF vs. control) as a fixed factor and AL and IOP as covariates (Type III sum of squares).

### 3.2. Intraocular Pressure (IOP)

After adjustment for AL, IOP was significantly higher in the CF group compared with controls (*p* = 0.007). AL was not a significant covariate (*p* = 0.272).

Adjusted means were 17.50 mmHg (95% CI 16.60–18.41) in the CF group and 15.87 mmHg (95% CI 15.19–16.55) in the control group. No significant change in IOP was observed following CFTR modulator treatment (*p* = 0.828).

### 3.3. Retinal Nerve Fiber Layer Thickness (RNFL)

**Figure 2:**
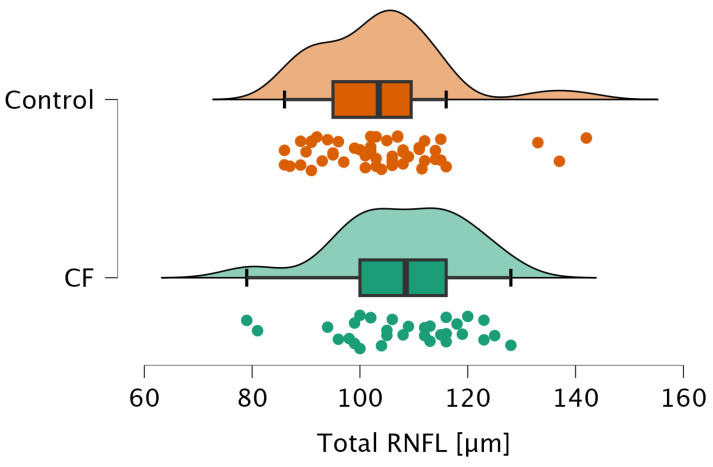
Comparison of total thickness of the retinal nerve fiber layer (TT RNFL) between cystic fibrosis (CF) patients and healthy controls. No significant between-group differences were observed after adjustment for axial length (AL) and intraocular pressure (IOP).

Total RNFL thickness did not differ significantly between the CF and control groups after adjustment for AL and IOP (*p* = 0.805) (Figure 2). However, AL was strongly associated with total RNFL thickness (*p* < 0.001), while IOP was not (*p* = 0.799). Descriptively, the median total RNFL thickness was 106.5 µm (IQR 15.25) in the CF group at baseline and 103.0 µm (IQR 14.0) in controls. No significant differences were observed for sectoral RNFL measures after adjustment for AL and IOP.

Longitudinally, total RNFL thickness decreased significantly following CFTR modulator therapy (*p* < 0.001), from a median of 106.5 µm (IQR 15.25) at baseline to 102.0 µm (IQR 15.5) at follow-up. No significant changes were detected for temporal RNFL thickness (74.5 µm vs. 71.5 µm, *p* = 0.813) or for the RNFL inferior-superior difference (1.0 vs. 1.0, *p* = 0.776). In contrast, significant reductions were observed in the superior quadrant (134.0 µm vs. 133.5 µm, *p* = 0.007), inferior quadrant (137.5 µm vs. 133.5 µm, *p* = 0.011), and nasal quadrant (80.0 µm vs. 78.5 µm, *p* = 0.038) over follow-up during CFTR modulator therapy.

No significant effect of CFRD status was observed for any RNFL parameter, including total and sectoral thickness (all *p* > 0.28).

### 3.4. Lamina Cribrosa Depth and Central Lamina Cribrosa Thickness

LCD did not significantly differ between the CF group and controls after adjustment for AL and IOP (*p* = 0.099). Neither AL nor IOP had a significant effect on LCD.

**Figure 3:**
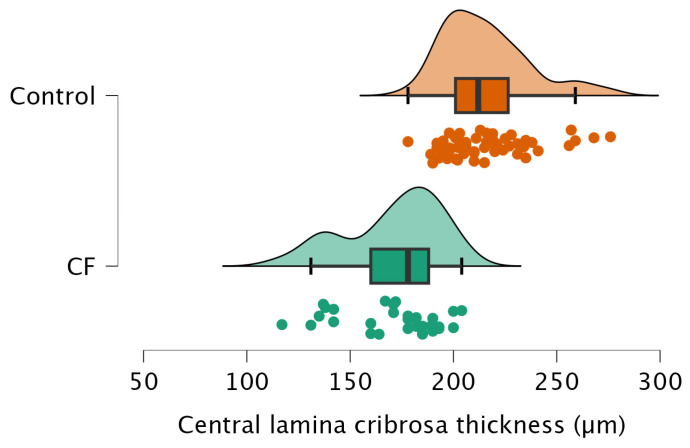
Comparison of central lamina cribrosa thickness (LCT) between cystic fibrosis (CF) patients and controls. CF individuals demonstrated significantly reduced central LCT.

After adjustment for AL and IOP (both not having a significant influence), the estimated marginal mean of central LCT was 168.4 µm (95% CI: 159.8–177.1) in the CF group and 216.7 µm (95% CI: 210.5–223.0) in the control group, indicating that the LC was thinner in CF (*p* < 0.001) (Figure 3).

No significant longitudinal change was observed following CFTR modulator therapy for either central LCT (*p* = 0.364) or LCD (*p* = 0.660). The median LCD was 425.5 µm (IQR 111.75) at baseline and 409.5 µm (IQR 188.75) at follow-up, whereas the median central LCT was 167.0 µm (IQR 51.0) at baseline and 168.5 µm (IQR 51.0) at follow-up.

In subgroup analysis for LCD, a difference was noted between controls and CF patients without diabetes (*p* = 0.016); however, this was driven by differences between controls and CF patients without diabetes, while no significant differences were found between CF patients with and without CFRD.

LCT measurements (central, mid-superior, and mid-inferior) did not differ between patients with and without CFRD (all *p* > 0.20), although both subgroups showed significantly lower values than controls (all *p* < 0.001).

### 3.5. Mid and Mean Lamina Cribrosa Thickness

Median LCT measured at the mid-superior and mid-inferior levels, as well as mean LC thickness, was significantly lower in patients with CF compared with healthy controls. For the mid-superior location, the median LC thickness was 151.5 µm (IQR 131.0–181.0) at the first examination and 163.0 µm (IQR 128.1–182.5) at follow-up, compared with 200.5 µm (IQR 192.0–214.5) in controls (both *p* < 0.001). Similarly, mid-inferior LC thickness was significantly lower in the CF group (155.8 µm [129.2–178.4] at baseline and 169.5 µm [124.2–179.0] at follow-up) than in controls (200.0 µm [187.1–207.5]; both *p* < 0.001). Mean LC thickness also differed significantly between groups, with lower values in patients with CF than in controls at both time points (*p* < 0.001). No significant longitudinal changes in LC thickness were observed between the first and second examination in the CF group.

### 3.6. Correlations

Correlation analyses were performed to explore associations between OCT-derived parameters and selected clinical variables in the CF group.

Correlation analyses in the CF group revealed a significant inverse association between total thickness of the RNFL and AL (Pearson r = −0.348, *p* = 0.032). AL was also negatively correlated with superior (r = −0.339, *p* = 0.038) and inferior (r = −0.512, *p* = 0.001) RNFL quadrant thickness. LCD showed a weak positive correlation with AL (r = 0.324, *p* = 0.047). Central LCT was not significantly correlated with AL. No significant correlations were observed between the total RNFL thickness and LC parameters (LCD and LCT). In the CF group, no significant associations were found between FEV_1_ and OCT-derived structural parameters. In the CF group, IOP showed a weak negative correlation with superior RNFL thickness (r = −0.294, *p* = 0.028). No significant correlations were found between IOP and AL, LCD, or LCT parameters (all *p* > 0.05).

## 4. Discussion

Ocular involvement in CF has received increasing attention in recent years, particularly with the wider availability of OCT imaging. Previous ophthalmological studies in CF have evaluated ocular surface parameters using the Schirmer test and non-invasive tear break-up time (N-TBUT), as well as anterior and posterior segment parameters using OCT and complementary imaging methods [11,12,14,15]. Previous research has focused on ONH measurements, such as peripapillary RNFL thickness [11,12], and, in some reports, GCC parameters [14] or other macular measurements [15]. While deeper ONH structures, such as the LC, have not been specifically evaluated in CF despite their recognized importance in ONH biomechanics and vascular regulation [5,16].

In this study, we aimed to provide a detailed assessment of ONH morphology in CF using SS-OCT. In addition, we investigated changes in analyzed parameters after 12–18 months of HEMT in CF group. It should be noted that HEMT has been associated with ocular side effects, most notably lens opacification [17]. However, the potential impact of HEMT on intraocular pressure and superficial and deep optic nerve structures has not been investigated.

Previous studies investigating ocular changes in CF have reported sectoral RNFL abnormalities. Giannakouras et al. found significantly lower nasal-inferior pRNFL thickness in adults with CF compared with controls (82 µm [IQR 67–102] vs. 92.5 µm [IQR 82–107], *p* = 0.005) [11], while Gutiérrez et al. reported reduced pRNFL thickness in the superior temporal sector (*p* = 0.002) [12]. In contrast, Saegebrecht et al. stratified CF patients into subgroups based on glucose metabolism abnormalities and observed more subtle macular RNFL differences. Specifically, parafoveal RNFL decreased from 23.21 µm (19.61–28.88) in normoglycemic CF patients to 21.82 µm (19.53–25.46) in patients with abnormal glycemic control and 20.47 µm (17.86–25.50) in CFRD, while perifoveal RNFL declined from 37.56 µm (29.08–49.68) to 35.31 µm (29.10–39.14) and 34.77 µm (26.29–49.71), respectively. Foveal RNFL showed significant negative correlations with glycemic variability indices—mean of daily differences (MODD: R = −0.605) and mean amplitude of glycemic excursions (MAGE: R = −0.609; both *p* < 0.05 [14]. Our results showed that RNFL differences disappeared after adjustment for AL. However, a direct comparison of previous studies’ results with our findings should be interpreted with caution due to differences in methodology. Previous studies compared CF adults with healthy controls using refractive error-based ocular characterization, and AL was not included as a covariate nor explored as a determinant of RNFL measurements. In contrast, our study shows that both total and sectoral RNFL thickness are strongly influenced by AL in CF patients, and that between-group differences in RNFL do not persist after appropriate biometry and IOP adjustment. The strong association between RNFL thickness and AL has been demonstrated in previous studies [18,19]. Therefore, we believe that the absence of between-group differences after adjustment indicates that previously reported RNFL alterations in CF may have been confounded by ocular biometry. CFRD was included as a testing variable due to the established association between diabetes and retinal neurodegeneration, including RNFL thinning, even in the absence of diabetic retinopathy or in its early stages [20,21]. However, in our cohort, no significant association between CFRD and RNFL thickness was observed.

Although our results showed longitudinal reduction in RNFL thickness in CF individuals, this finding should be interpreted with caution. The absence of cross-sectional differences after AL adjustment and the lack of parallel changes in deep ONH structures suggests that the underlying mechanisms may extend beyond a primary retinal neurodegenerative process. Instead, these changes may reflect systemic factors such as alterations in hydration status, vascular regulation, or inflammatory activity [6,22]. While a subtle neuroaxonal component cannot be entirely excluded, the overall pattern of findings does not support progressive optic nerve degeneration.

We found consistent alterations in deep ONH structure in CF group—reduced LC thickness—which was independent of AL and IOP. This indicates that CF may preferentially affect the deep connective tissue structure of the ONH, particularly LC—a structure that has not been analyzed in previous studies in individuals with CF. We believe that our results suggest that LCT may represent potential structural marker of systemic involvement in CF, independent of ocular biometry; however, this observation should be considered preliminary and requires confirmation in larger, longitudinal studies.

LC is highly sensitive to systemic diseases associated with hypoxia, vascular dysregulation, and chronic metabolic and inflammatory factors, as it is a biomechanically active connective tissue structure that is exposed to alterations in the translaminar pressure gradient and is dependent on a delicate microvascular supply [5,7].

Interestingly, the characteristics of LC changes observed in our CF cohort partly resembles those reported in glaucomatous eyes. In glaucoma, the LC is typically described as thinner and more posteriorly displaced than in healthy controls [23,24]. In our study, CF individuals likewise demonstrated reduced LCT. However, unlike glaucoma, LC thinning was not accompanied by increased LCD and independent RNFL thinning after adjustment for AL and IOP, indicating that the LC phenotype in CF may reflect systemic connective tissue, vascular, or hypoxia-related stress rather than a classic glaucomatous pattern. Therefore, the present findings do not establish a glaucoma-like pathogenic mechanism, and the observed similarity should be interpreted with caution.

Deep ONH structural changes have also been described in a variety of systemic diseases. Ermiş et al. demonstrated alterations in LC configuration in patients with obstructive sleep apnea syndrome (OSAS), a condition characterized by recurrent nocturnal hypoxia. OSAS patients showed increased curvature of the posterior LC surface, and higher apnea–hypopnea index values were associated with thinner LC, demonstrating that hypoxic stress may be linked to LC remodeling [25]. Similar to hypoxic abnormalities, vascular disorders affecting ONH perfusion have also been associated with LC structural changes. In non-arteritic anterior ischemic optic neuropathy (NAION), Rebolleda et al. showed dynamic changes in LC position relative to the Bruch’s membrane opening, with backward LC displacement at the acute stage, followed by significant forward displacement during follow-up, along with progressive BMO changes [26]. In a subsequent cross-sectional study, a more anteriorly placed LC in NAION and a more deeply placed LC in eyes with POAG than in healthy controls were reported [27], supporting the concept that impaired ONH perfusion may influence laminar position.

We did not find significant differences in LC parameters between CF individuals with and without CFRD, implying that the observed thinning of the LC is related to CF rather than mediated by CFRD. In non-CF diabetic patients, Akkaya et al. found that the LC was thicker and located more anteriorly than in healthy controls, indicating that systemic metabolic or microvascular factors may alter laminar remodeling and connective tissue properties [28]. Our findings demonstrate that deep ONH alterations are independent of CFRD status and shows that LC structure may reflect the effects of systemic hypoxic, vascular, and inflammatory stress. In this context, the reduced LCT observed in the present study may reflect a structural response of the ONH to chronic systemic CF-related factors, although this interpretation remains hypothesis.

In the CF group, IOP showed a weak negative correlation with superior RNFL thickness, while no significant associations were observed with LC parameters, AL, or other ONH measurements. Although IOP was significantly higher in CF group, the absence of consistent correlations with LC parameters and most ONH measures implies that the observed structural alterations may reflect systemic CF-related mechanisms rather than IOP-secondary remodeling.

The present study provides new insights into ONH morphology in adult CF patients using SS-OCT. While peripapillary RNFL thickness did not differ significantly between patients with CF and healthy controls after adjustment for AL and IOP, thinner LC was observed in patients with CF compared with controls. This finding suggests that structural changes in CF may primarily involve the deep connective tissue of the ONH rather than the superficial RNFL. Further studies are necessary to determine if changes at the LC level may precede detectable alterations in superficial retinal layers in CF patients.

This study has several limitations that should be considered. The relatively small sample size may limit the statistical power of the findings. Due to technical constraints, manual analysis of ONH structures was limited to a single central B-scan, yielding a localized rather than a fully three-dimensional assessment. BMO and LC borders were manually identified; therefore, this may account for researcher variability. To minimize this effect, only scans with clearly identifiable anatomical landmarks were included.

A strength of this study is the explicit consideration of ocular biometry, particularly AL, in the analysis of superficial and deep ONH parameters, thereby reducing biometry-related confounding and enabling a more independent interpretation of OCT findings. Our study also evaluates the potential impact of CFRD on investigated parameters, an issue not previously addressed. In addition, longitudinal follow-up after 12–18 months of CFTR modulator therapy provides novel insights into the stability of both superficial and deep ONH parameters during CF treatment.

## 5. Conclusions

Our study revealed that RNFL differences in CF do not persist after adjustment for AL and IOP, underscoring the importance of ocular biometry and IOP in interpreting OCT-derived RNFL measurements. In contrast, patients with CF exhibited a thinner LC, independent of AL and IOP, suggesting that CF alterations may involve the deep ONH architecture; however, these findings should be interpreted with caution and require validation in larger, prospective studies. Although IOP was higher in CF group, its limited association with structural parameters implies that it is unlikely to be a primary driver of the observed ONH alterations; however, this finding warrants further investigation. The LC phenotype was not explained by CFRD, supporting the conclusion that our findings reflect a structural feature of CF rather than a secondary metabolic disturbance.

## Figures and Tables

**Table 1:** Demographic, systemic, and OCT-related baseline characteristics of the studied groups.

Parameter	CF (*n* = 34)	Controls (*n* = 34)	*p*-Value
Age, years	28.0 (22.0–33.0) *	26.0 (26.0–30.0) *	0.619
Sex, *n* (%) female	23 (67.6%)	23 (67.6%)	1.000
Spherical equivalent, D ^	−0.38 (−1.66–0.12) *	−0.75 (−2.50–0.00) *	0.308
Axial length, mm	22.80 ± 1.03	23.82 ± 1.15	0.002
Intraocular pressure, mmHg	17.214 ± 2.172	16.067 ± 2.544	0.011
FEV_1_, % predicted	59.5 ± 19.0	—	—
Tiffeneau index (FEV_1_/FVC)	78.1 ± 12.0	—	—
CF-related diabetes (CFRD), *n* (%)	9 (28.1%)	—	—

Data are presented as mean ± standard deviation unless otherwise indicated. * data are presented as median and IQR. Between-group comparison: CF at baseline vs. healthy controls. ^ Refractive status was assessed using spherical equivalent, and participants with high refractive error were excluded according to predefined criteria.

## Data Availability

The data are available from the corresponding author on reasonable request.

## Supplementary Materials

The following supporting information can be downloaded at: https://www.mdpi.com/article/10.3390/jcm15114308/s1, Supplementary File. Table S1: Comparison of OD vs OS in CF Group (normally distributed variables, Student’s *t*-test). Table S2: Comparison of OD vs OS in CF Group (non-normally distributed variables, Mann-Whitney U test). Table S3: Comparison of OD vs OS in Control Group (normally distributed variables, Student’s *t*-test). Table S4: Comparison of OD vs OS in Control Group (non-normally distributed variables, Mann-Whitney U test).

## Abbreviations

The following abbreviations are used in this manuscript:

AL	Axial length
ANCOVA	Analysis of covariance
BCVA	Best-corrected visual acuity
BMO	Bruch’s membrane opening
CF	Cystic fibrosis
CFRD	CF-related diabetes
CFTR	Cystic fibrosis transmembrane conductance regulator
CI	Confidence interval
CNS	Central nervous system
FEV_1_	Forced expiratory volume in one second
IOP	Intraocular pressure
IQR	Interquartile range
LC	Lamina cribrosa
LCD	Lamina cribrosa depth
LCT	Lamina cribrosa thickness
MAGE	Mean amplitude of glycemic excursions
MODD	Mean of daily differences
OCT	Optical coherence tomography
ONH	Optic nerve head
RNFL	Retinal nerve fiber layer
SD	Standard deviation
SD-OCT	Spectral-domain optical coherence tomography
SS-OCT	Swept-source optical coherence tomography

## References

[1] Kerem E., Orenti A., Adamoli A., Hatziagorou E., Naehrlich L., Sermet-Gaudelus I., the ECFS Patient Registry Steering Group (2024). Cystic fibrosis in Europe: Improved lung function and longevity-reasons for cautious optimism, but challenges remain. Eur. Respir. J..

[2] McBennett K.A., Davis P.B., Konstan M.W. (2022). Increasing life expectancy in cystic fibrosis: Advances and challenges. Pediatr. Pulmonol..

[3] Shah P.H., Lee J.H., Salvi D.J., Rabbani R., Gavini D.R., Hamid P. (2021). Cardiovascular System Involvement in Cystic Fibrosis. Cureus.

[4] Liberski S., Confalonieri F., Cofta S., Petrovski G., Kocięcki J. (2024). Ocular Changes in Cystic Fibrosis: A Review. Int. J. Mol. Sci..

[5] Burgoyne C.F., Crawford Downs J., Bellezza A.J., Francis Suh J.-K., Hart R.T. (2005). The optic nerve head as a biomechanical structure: A new paradigm for understanding the role of IOP-related stress and strain in the pathophysiology of glaucomatous optic nerve head damage. Prog. Retin Eye Res..

[6] Vujosevic S., Parra M.M., Hartnett M.E., O’tOole L., Nuzzi A., Limoli C., Villani E., Nucci P. (2023). Optical coherence tomography as retinal imaging biomarker of neuroinflammation/neurodegeneration in systemic disorders in adults and children. Eye.

[7] Paulo A., Vaz P.G., De Jesus D.A., Brea L.S., Van Eijgen J., Cardoso J., van Walsum T., Klein S., Stalmans I., Breda J.B. (2021). Optical Coherence Tomography Imaging of the Lamina Cribrosa: Structural Biomarkers in Nonglaucomatous Diseases. J. Ophthalmol..

[8] Ergen A., Tuğan B.Y. (2025). Lamina cribrosa curvature depth and index as novel parameters in Graves’ ophthalmopathy. Sci. Rep. Nat. Publ. Group.

[9] Cunha L.P., Pires L.A., Cruzeiro M.M., Almeida A.L.M., Martins L.C., Martins P.N., Shigaeff N., Vale T.C. (2022). Optical coherence tomography in neurodegenerative disorders. Arq. Neuropsiquiatr..

[10] Nuyen B., Mansouri K., Weinreb R.N. (2012). Imaging of the Lamina Cribrosa using Swept-Source Optical Coherence Tomography. J. Curr. Glaucoma Pract..

[11] Giannakouras P., Kanakis M., Diamantea F., Tzetis M., Koutsandrea C., Papaconstantinou D., Georgalas I. (2021). Ophthalmologic manifestations of adult patients with cystic fibrosis. Eur. J. Ophthalmol..

[12] Gutiérrez P., Jiménez L., Martínez J., Alba C., Girón M.V., Olveira G., Ruiz-Esteban P., Olveira C. (2025). Dry eye disease and morphological changes in the anterior chamber in people with cystic fibrosis. J. Cyst. Fibros..

[13] Weathered N.R. (2020). Cardiac and Pulmonary Disorders and the Nervous System. Continuum.

[14] Saegebrecht L.S., Röhlig M., Schaub F., Ballmann M., Stachs O., Fischer D.-C. (2024). Glycemic Variability and the Thickness of Retinal Layers in Cystic Fibrosis Patients with and without Cystic Fibrosis Related Diabetes. Curr. Eye Res..

[15] Shi A.J., Morrissey B.M., Durbin-Johnson B., Pilli S., Zawadzki R.J., Cross C.E., Park S.S. (2014). Macular pigment and macular volume in eyes of patients with cystic fibrosis. Free Radic. Res..

[16] Karimi A., Rahmati S.M., Grytz R.G., Girkin C.A., Downs J.C. (2021). Modeling the biomechanics of the lamina cribrosa microstructure in the human eye. Acta Biomater..

[17] Schneider-Futschik E.K., Zhu Y., Li D., Habgood M.D., Nguyen B.N., Pankonien I., Amaral M.D., Downie L.E., Chinnery H.R. (2024). The role of CFTR in the eye, and the effect of early highly effective modulator treatment for cystic fibrosis on eye health. Prog. Retin. Eye Res..

[18] Savini G., Barboni P., Parisi V., Carbonelli M. (2012). The influence of axial length on retinal nerve fibre layer thickness and optic-disc size measurements by spectral-domain OCT. Br. J. Ophthalmol..

[19] Wang S.V., Li N., Rice D.S., Grosskreutz C.L., Dryja T.P., Prasanna G., Lii J., Gagne J.J. (2019). Using Healthcare Databases to Refine Understanding of Exploratory Associations Between Drugs and Progression of Open-Angle Glaucoma. Clin. Pharmacol. Ther..

[20] Cipres Alastuey M., Gavin Sancho A., Satue Palacian M., Rodrigo Sanjuan M., Orduna Hospital E., Vilades Palomar E., Larrea Samper J.G., Garcia Martin E. (2018). Retinal measurements in type 2 diabetic patients without diabetic retinopathy using Swetpt-Source Optical coherence tomography Triton device. Acta Ophthalmol..

[21] Bhaskaran A., Babu M., Sudhakar N.A., Kudlu K.P., Shashidhara B.C. (2023). Study of retinal nerve fiber layer thickness in diabetic patients using optical coherence tomography. Indian J. Ophthalmol..

[22] İnam O., Kaplan H.J., Tezel T.H. (2023). Retinal Hydration Assessment With Optical Coherence Tomography: Unraveling Its Significance in Retinal Fluid Dynamics, Macular Edema and Cell Viability. Transl. Vis. Sci. Technol..

[23] Furlanetto R.L., Park S.C., Damle U.J., Sieminski S.F., Kung Y., Siegal N., Liebmann J.M., Ritch R. (2013). Posterior displacement of the lamina cribrosa in glaucoma: In vivo interindividual and intereye comparisons. Invest Ophthalmol. Vis. Sci..

[24] Park H.-Y.L., Jeon S.H., Park C.K. (2012). Enhanced depth imaging detects lamina cribrosa thickness differences in normal tension glaucoma and primary open-angle glaucoma. Ophthalmology.

[25] Ermis S., Ozal E., Arabaci I.C., Hos U.D., Gul C., Karapapak M., Ozal S.A. (2025). Lamina cribrosa curvature index as a biomarker of non-glaucomatous optic neuropathy in obstructive sleep apnea syndrome. Indian J. Ophthalmol..

[26] Rebolleda G., García-Montesinos J., De Dompablo E., Oblanca N., Muñoz-Negrete F.J., González-López J.J. (2017). Bruch’s membrane opening changes and lamina cribrosa displacement in non-arteritic anterior ischaemic optic neuropathy. Br. J. Ophthalmol..

[27] Rebolleda G., Pérez-Sarriegui A., Díez-Álvarez L., De Juan V., Muñoz-Negrete F.J. (2019). Lamina cribrosa position and Bruch’s membrane opening differences between anterior ischemic optic neuropathy and open-angle glaucoma. Eur. J. Ophthalmol..

[28] Akkaya S., Küçük B., Doğan H.K., Can E. (2018). Evaluation of the lamina cribrosa in patients with diabetes mellitus using enhanced depth imaging spectral-domain optical coherence tomography. Diab Vasc. Dis. Res..

