# Supplementary material for: Deep Optic Nerve Head Structural Alterations in Adults with Cystic Fibrosis
Source: J Clin Med. 2026 Jun 2;15(11):4308. doi: 10.3390/jcm15114308 (PMC13258307; doi:10.3390/jcm15114308)
Supplement: Supplementary file 1 [file jcm-15-04308-s001.zip › jcm-4256540-supplementary.pdf]

Suplement file (Supplementary table S1-S4) - intrapersonal analysis of the variables.

Supplementary table S1. Comparison of OD vs OS in CF Group (normally distributed variables, Student's t-test)

	t	df	p
IOP	0.228	114	.820
RNFL S	-0.836	131	.405
RNFL I	-0.097	131	.923
TT RNFL	0.451	131	.653
RNFL	0.585	131	.560
Lamina cribrosa depth	-0.213	133	.831

Supplementary table S2. Comparison of OD vs OS in CF Group (non-normally distributed variables, Mann-Whitney U test)

	U	p
AL	1,220.5	.890
S	2,022.0	.979
C	2,026.5	.961
A	1,904.5	.591
SE	1,991.0	.853
RNFL T	2,469.5	.245
RNFL diff I-S	2,345.0	.655
Lamina cribrosa thickness – central (μm)	1,967.0	.934
Lamina cribrosa thickness –mid S (μm)	1,804.5	.474
Lamina cribrosa thickness –mid I (μm)	1,401.5	.068
Mean lamina cribrosa thickness (μm)	2,091.5	.413

Supplementary table S3. Comparison of OD vs OS in Control Group (normally distributed variables, Student's t-test)

	t	df	p
SE	0.165	63	.869
IOP	-0.101	58	.920
RNFL S	-0.628	63	.532
RNFL I	-0.417	63	.678
RNFL T	0.578	63	.565
RNFL	0.774	63	.442
RNFL diff I-S	0.145	64	.885
Lamina cribrosa depth (μm)	0.191	65	.849
Lamina cribrosa thickness –mid S (μm)	-1.331	61	.188
Lamina cribrosa thickness –mid I (μm)	-1.719	59	.091
Mean lamina cribrosa thickness (μm)	-1.364	65	.177

Supplementary table S4. Comparison of OD vs OS in Control Group (non-normally distributed variables, Mann-Whitney U test)

	U	p
AL	453.0	.971
S	578.5	.831
C	566.5	.949
A	501.0	.453
TT RNFL	545.0	.828
Lamina cribrosa thickness – central (μm)	470.0	.256
